# Supplementary material for: Specific sensory neurons and insulin-like peptides modulate food type-dependent oogenesis and fertilization in Caenorhabditis elegans
Source: eLife. 2023 Nov 17;12:e83224. doi: 10.7554/eLife.83224 (PMC10665013; doi:10.7554/eLife.83224)
Supplement: Supplementary file 1. [file elife-83224-supp1.docx]

**Supplementary File 1. List of strains**

| **Strain** | **Genotype** | **Reference(s)** |
| --- | --- | --- |
| **Oogenesis marker, *lin-41::GFP*** | *lin-41(tn1541[GFP::tev::s::lin-41])* | Spike et al., 2014 |
| **Oogenesis marker, *oma-1::GFP*** | *unc-119(ed3); teIs1(oma-1::GFP + unc-119[+])* | Lin, 2003 |
| **Sensory mutants** | *odr-1(n1936)* | L'Etoile and Bargmann, 2000 |
|  | *odr-3(n1605)* | Roayaie et al., 1998 |
|  | *odr-10(ky225)* | Sengupta et al., 1996 |
|  | *osm-3(p802)* | Tabish et al., 1995 |
|  | *lin-41(tn1541); osm-3(p802)* | This work |
| **ADF (-)** | *udEx211(srh-142::ced-3(p15); srh-142::ced-3(p17;*  *srh-142::gfp; elt-2::gfp)* | Krzyzanowski et al., 2016 |
| **ASH (-)** | *peIs1713(sra-6p::mCasp-1 + unc-122p::mCherry)* | Yoshida et al., 2012 |
| **ASI (-)** | *oyIs84(gpa-4p::TU813 gcy-27p::TU814 gcy-*  *27p::GFP coelp::dsRED)* | Beverly et al., 2011 |
|  | *lin-41(tn1541); oyIs84* | This work |
| **ASJ (-)** | *pgIs(trx-1p::ICE)* | Cornils et al., 2011; integrated by M.  Goodman (gift) |
|  | *lin-41(tn1541); pgIs(trx-1p::ICE)* | This work |
|  | *teIs1(oma-1::GFP + unc-119[+]); pgIs(trx-1p::ICE)* | This work |
| **ASK (-)** | *qrls2(sra-9::mCasp1)* | Srinivasan et al., 2012 |
| **AWA (-)** | *odr-7(ky4)* | Sengupta et al., 1994 |
|  | *lin-41(tn1541); odr-7(ky4)* | This work |
|  | *Ex[odr-10::mCasp1 + myo-3::GFP]* | Yoshida et al., 2012; Schiffer et al.,  2020 |
| **AWB (-)** | *peIs1715(str-1p::mCasp-1 + unc-122p::GFP)* | Yoshida et al., 2012 |
| **AWC (-)** | *oyIs85(ceh-36p::TU#813 + ceh-36p::TU#814 + srtx-*  *1p::GFP + unc-122p::DsRed)* | Beverly et al., 2011 |
| **ASJ (-); AWA(-)** | *pgIs(trx-1p::ICE); odr-7(ky4)* | This work |
|  | *lin-41(tn1541); pgIs(trx-1p::ICE); odr-7(ky4)* | This work |
| **Insulin signaling mutants** | *daf-2(e1368)* | Gems et al., 1998 |
|  | *lin-41(tn1541); daf-2(e1368)* | This work |
|  | *daf-2(e1370)* | Gems et al., 1998 |
|  | *lin-41(tn1541); daf-2(e1370)* | This work |
|  | *daf-16(mu86)* | Lin et al., 1997 |
|  | *lin-41(tn1541) daf-16(mu86)* | This work |
|  | *ins-1(nr2091)* | Pierce et al., 2001 |
|  | *lin-41(tn1541); ins-1(nr2091)* | This work |
|  | *ins-6(tm2416)* | Cornils et al., 2011 |
|  | *lin-41(tn1541); ins-6(tm2416)* | This work |
|  | *lin-41(tn1541) daf-16(mu86); ins-6(tm2416)* | This work |
|  | *ins-6(tm2416); teIs1(oma-1::GFP + unc-119[+])* | This work |
|  | *daf-28(tm2308)* | Cornils et al., 2011 |
|  | *lin-41(tn1541); daf-28(tm2308)* | This work |
|  | *ins-6(tm2416); daf-28(tm2308)* | Cornils et al., 2011 |
|  | *ins-6(tm2416); ins-1(nr2091)* | Cornils et al., 2011 |
|  | *ins-1(nr2091); daf-28(tm2308)* | Cornils et al., 2011 |
|  | *ins-6(tm2416); ins-1(nr2091); daf-28(tm2308)* | Cornils et al., 2011 |
| ***ins-6* full genomic rescue** | *lin-41(tn1541); ins-6(tm2416); jxEx27(ins-6p::ins-6 [2*  *ng/ul]; ofm-1::GFP [25 ng/ul])* | Cornils et al., 2011; this work |
|  | *lin-41(tn1541); ins-6(tm2416); jxEx28(ins-6p::ins-6 [2*  *ng/ul]; ofm-1::GFP [25 ng/ul])* | Cornils et al., 2011; this work |

**Supplementary File 1. List of strains (continued)**

| **Strain** | **Genotype** | **Reference(s)** |
| --- | --- | --- |
| ***ins-6* ASI-specific rescue** | *lin-41(tn1541); ins-6(tm2416); jxEx51(str-3p::ins-6*  *[25 ng/ul]; ofm-1::GFP [25 ng/ul])* | Cornils et al., 2011; this work |
| ***ins-6* ASJ-specific rescue** | *lin-41(tn1541); ins-6(tm2416); jxEx58(trx-1p::ins-6*  *[25 ng/ul]; ofm-1::GFP [25 ng/ul])* | Cornils et al., 2011; this work |
|  | *lin-41(tn1541); ins-6(tm2416); jxEx59(trx-1p::ins-6*  *[25 ng/ul]; ofm-1::GFP [25 ng/ul])* | Cornils et al., 2011; this work |
| ***ins-6p::mCherry drcSi68*** | *ttTi5605 drcSi68(ins-6p::mCherry; Cb-unc-119[+])II* | This work |
| **floxed *ins-6* locus** | *lin-41(tn1541); ins-6(syb7547)* | This work |
| **ASJ-specific *nCRE*** | *lin-41(tn1541); ins-6(tm2416); jxEx225(trx- 1p::nCRE::unc-54 3’UTR [50 ng/ul] + unc-122p::GFP*  *[25 ng/ul])* | This work |

**Supplementary references**

Beverly, M., Anbil, S., and Sengupta, P. 2011. Degeneracy and neuromodulation among thermosensory neurons contribute to robust thermosensory behaviors in *Caenorhabditis elegans*. *J Neurosci* **31**: 11718-11727.

Cornils, A., Gloeck, M., Chen, Z., Zhang, Y., and Alcedo, J. 2011. Specific insulin-like peptides encode sensory information to regulate distinct developmental processes. *Development* **138:** 1183-1193.

Gems, D., Sutton, A.J., Sundermeyer, M.L., Albert, P.S., King, K.V., Edgley, M.L., Larsen, P.L., and Riddle, D.L. 1998. Two pleiotropic classes of *daf-2* mutation affect larval arrest, adult behavior, reproduction and longevity in *Caenorhabditis elegans*. *Genetics* **150:** 129-155.

Krzyzanowski, M.C., Woldemariam, S., Wood, J.F., Chaubey, A.H., Brueggemann, C., Bowitch, A., Bethke, M., L’Etoile, N.D., and Ferkey, D.M. 2016. Aversive behavior in the nematode *C. elegans* is modulated by cGMP and a neuronal gap junction network. *PLoS Genet* **12:** e1006153.

L'Etoile, N.D., and Bargmann, C.I. 2000. Olfaction and odor discrimination are mediated by the

*C. elegans* guanylyl cyclase ODR-1. *Neuron* **25:** 575-586.

Lin, K., Dorman, J.B., Rodan, A., and Kenyon, C. 1997. *daf-16*: An HNF-3/forkhead family member that can function to double the life-span of *Caenorhabditis elegans*. *Science* **278:** 1319–1322.

Lin R. 2003. A gain-of-function mutation in *oma-1*, a *C. elegans* gene required for oocyte maturation, results in delayed degradation of maternal proteins and embryonic lethality, *Dev Biol* **258**: 226-239.

Pierce, S.B., Costa, M., Wisotzkey, R., Devadhar, S., Homburger, S.A., Buchman, A.R., Ferguson, K.C., Heller, J., Platt, D.M., Pasquinelli, A.A.*, et al.* 2001. Regulation of DAF-2 receptor signaling by human insulin and *ins-1*, a member of the unusually large and diverse *C. elegans* insulin gene family. *Genes Dev* **15:** 672-686.

Roayaie, K., Crump, J.G., Sagasti, A., and Bargmann, C.I. 1998. The G alpha protein ODR-3 mediates olfactory and nociceptive function and controls cilium morphogenesis in *C. elegans* olfactory neurons. *Neuron* **20:** 55-67.

Schiffer, J.A.*,* Servello, F.A., Heath, W.R., Amrit, F.R.G., Stumbur, S.V., Eder, M., Martin, O.M.F., Johnsen, S.B., Stanley, J.A., Tam, H.*, et al.* 2020. *Caenorhabditis elegans* processes sensory information to choose between freeloading and self-defense strategies. *eLife* **9:** e56186.

Sengupta, P., Chou, J.H., and Bargmann, C.I. 1996. *odr-10* encodes a seven transmembrane domain olfactory receptor required for responses to the odorant diacetyl. *Cell* **84:** 899-909.

Sengupta, P., Colbert, H.A., and Bargmann, C.I. 1994. The *C. elegans* gene *odr-7* encodes an olfactory-specific member of the nuclear receptor superfamily. *Cell* **79:** 971-980.

Spike, C.A., Coetzee, D., Eichten, C., Wang, X., Hansen, D., and Greenstein, D. 2014. The TRIM-NHL protein LIN-41 and the OMA RNA-binding proteins antagonistically control the prophase-to-metaphase transition and growth of *Caenorhabditis elegans* oocytes. *Genetics* **198:** 1535-1558.

Srinivasan, J., von Reuss, S.H., Bose, N., Zaslaver, A., Mahanti, P., Ho, M.C., O'Doherty, O.G., Edison, A.S., Sternberg, P.W., and Schroeder, F.C. 2012. A modular library of small molecule signals regulates social behaviors in *Caenorhabditis elegans*. *PLoS Biol* **10:** e1001237.

Tabish, M., Siddiqui, Z.K., Nishikawa, K., and Siddiqui, S.S. 1995. Exclusive expression of *C. elegans osm-3* kinesin gene in chemosensory neurons open to the external environment. *J Mol Biol* **247:** 377-389.

Yoshida, K., Hirotsu, T., Tagawa, T., Oda, S., Wakabayashi, T., Iino, Y., and Ishihara, T. 2012. Odour concentration-dependent olfactory preference change in *C. elegans*. *Nat commun* **3:** 739.
